# How the manner in which data is visualized affects and corrects (mis)perceptions of political polarization

**DOI:** 10.1111/bjso.12787

**Published:** 2024-07-17

**Authors:** JonRobert Tartaglione, Lee de‐Wit

**Affiliations:** ^1^ University of Cambridge Cambridge UK

**Keywords:** data visualization, intervention, misperception, polarization, politics

## Abstract

While the mechanisms underlying polarization are complex, scholars have consistently found a pervasive overestimation of *perceptions* of polarization to be a contributing factor. We argue that one mitigation strategy that can work at scale to address such misperceptions might be relatively straightforward: better data visualizations of cross‐party attitudes on key issues. In a large‐scale (*N* = 6603), international replication, we find that *mode of presentation*—or the manner in which data are visually presented—plays a significant role in moderating perceptions of polarization, even for longstanding, divisive issues for which partisans would likely hold strong prior beliefs. Additionally, we find the effects that different modes of presentation have on issue‐specific polarization also extend to participant beliefs about *overall* interparty polarization, with certain modes proving capable of not only promoting less polarized views but also enabling more accurate estimates of the extent to which political groups agree. Finally, our findings also suggest that the manner in which intergroup data are visualized may also exert influence over the degree to which political groups are *essentialized—*a finding with implications for not only political perception but also for apolitical social psychological phenomena such as dehumanization.

## INTRODUCTION

For years, affective and ideological polarization has been increasing across the United States and certain other Western democracies (Druckman & Levy, 2022; Garzia et al., 2023; McCarty et al., 2016; Pew Research Center, 2017). Rising levels of polarization in the US threaten effective legislative functioning (McCarty, 2016), reduce the willingness of partisans to engage with those outside their political ingroup (Baldassarri & Page, 2021; Fiorina & Abrams, 2008) and have the potential to exacerbate prejudice towards and dehumanization of political opponents (Cassese, 2021; Iyengar et al., 2012). Moreover, rising levels of polarization may reduce support for (Levendusky & Malhotra, 2016) or otherwise inhibit mutually beneficial compromises between political rivals (Whitt et al., 2021). However, while polarization is undoubtedly increasing in several metrics across the US, a growing number of scholars have begun to address the phenomenon known as *false polarization* (e.g. Fernbach & Van Boven, 2022), wherein individuals overestimate both the magnitude of disagreement in the political sphere as well as the ideological consistency of the actors operating within it.

Research has shown that partisans struggle to identify the actual position of their adversaries on key issues (Chambers et al., 2006) and often base their assumptions on stereotypic thinking which serves to exaggerate the differences between one's ingroup and outgroup (Graham et al., 2012; Keltner & Robinson, 1993). These propensities can yield profound ‘perception gaps’ (Yudkin et al., 2019), wherein distorted beliefs about where the other party stands on issues differs dramatically from the actual positions they hold. More broadly, Westfall et al. (2015) cite evidence showing that Americans have exhibited a pattern of overestimation of polarization which has spanned more than three decades. Consistent with such a finding, recent research has begun to focus on group meta‐perceptions, where similar inaccuracies continue to emerge (Lees & Cikara, 2020). Importantly, these results appear to extend well beyond the confines of the United States (Ruggeri et al., 2021).

While political misperceptions have important consequences for interparty relations (Ahler & Sood, 2018; Moore‐Berg et al., 2020), they may also be capable of engendering *actual* polarization through a process of mutual reinforcement (Lees & Cikara, 2021). Thus, it is important that such misperceptions are corrected whenever possible. Several studies have shown that interventions which expose individuals to the actual positions espoused by their own as well as opposing political groups can reduce negative outgroup attributions (Lees & Cikara, 2020) and promote political moderation (Ahler, 2014) via the attenuation of beliefs about attitudinal extremity.

However, Druckman and Levy (2022) note that individual interventions, though promising, might still struggle to overcome persistent misperceptions on a large scale due to the fact that many misperceptions are institutionally driven and sustained (see Wilson et al., 2020). More specifically, because exposure to (often exaggerated) polarization narratives is near‐ubiquitous in today's modern media landscape, often being promoted by agents with immense scope for influence (e.g. partisan media outlets, political elites, etc.), interventions seeking to effectively counter such influence should have the capacity to be deployed on an equally large scale, ideally with minimal ‘training’ or requirements of sustained attention on the part of the target audience. As data visualizations are already an omnipresent feature of print, television and social media, should certain visualizations demonstrate the ability to reduce political misperceptions, simply shifting current data presentation methodologies to these visualization formats would represent a promising avenue for catalyzing widespread change with minimal effort. Such an intervention would also have an excellent cost–benefit profile and superior scalability to individually‐tailored interventions, permitting depolarization agents to easily embed perception‐correction mechanisms across multiple domains and mediums in which visualizations already exist.

There are reasons to believe altering visualizations may be a viable mechanism through which one could shift perceptions of groups. Scholars like Hanel et al. (2019) have argued that traditional means of presenting data about groups—such as bar charts with truncated *y*‐axes—may (purposely or otherwise) accentuate group *differences* while ignoring equally important similarities. Modes of presentation like truncated bar charts provide little in the way of distributional information, which may inadvertently facilitate oversimplification and categorical thinking (cognitive mechanisms that contribute to false polarization; Fernbach & Van Boven, 2022). Moreover, an oversimplification of group beliefs coupled with exaggerated depictions of group differences may risk potentially facilitating more ‘essentialized’ perceptions of the depicted groups (see Haslam & Whelan, 2008), wherein individuals conceptualize social categories—and, consequently, the delineation between different groups—as both natural and fixed. Beliefs in ‘natural kinds’ have been found to amplify group differences (Rothbart & Taylor, 1992) and the attribution of ‘essences’ to outgroups has been linked to classic conceptions of prejudice (Allport et al., 1954). However, while the modes that have traditionally been used to visualize data may unintentionally magnify perceived group differences and lead to exaggerated beliefs about political polarization, Hanel et al. (2019) found that simply changing the type of visualization used to depict identical data had a significant impact across an array of intergroup assessments, with modes that more effectively highlight similarity information (e.g. superimposed normal distributions) resulting in both more accurate intergroup perceptions as well as more positive outgroup appraisals.

While such findings might seem modest, we believe they have the potential to be particularly impactful in the context of political polarization. Researchers have consistently shown that minute, sometimes seemingly inconsequential changes to the way in which data are visualized can profoundly alter how that data are perceived and interpreted (e.g. see Lo et al., 2022; Nguyen et al., 2021). Considering this, we believe that it is fair to question whether the manner in which we have *traditionally* visualized data about political groups may contribute to political misperceptions and false polarization. In addition to expounding upon the disadvantages of traditional modes of presentation and the possible dangers of *y*‐axis truncation in depicting group data, the results reported by Hanel et al. (2019) also provide preliminary evidence for the potential power of using comparatively novel visualization techniques (i.e. those which feature aspects such as full ranges and distributions of responses, levels of intergroup agreement as opposed to simply disagreement, etc.) to shift intergroup perceptions. We believe that this should motivate researchers to consider how expanding their data depiction methodologies beyond the traditional (e.g. bar charts) might similarly alter *political* perceptions. For example, modes such as icon arrays, which feature prominently in the risk communication literature, have shown promise in areas such as increasing the accuracy of risk perception (Galesic et al., 2009) and mitigating phenomena such as denominator neglect (Garcia‐Retamero et al., 2010),^1^ but have yet to be extensively explored in the context of polarization.

While other researchers have investigated the circumstances and contexts in which data visualizations might prove capable of changing attitudes (e.g. Markant et al., 2022; Pandey et al., 2014), few have directly focused on how certain depictions of data may affect beliefs about the extent of political polarization (Alieva, 2023; Santos et al., 2017). There is some precedence for believing that ‘better’ visualizations might constitute useful interventions to reduce political polarization. For example, Rutchick et al. (2009) found that maps depicting state‐level support for a presidential candidate with a shade of purple along a red and blue continuum (in proportion to the degree of support for the Republican or Democratic candidate) reduced stereotyping as well as perceptions of political division compared to the traditional binary red and blue state‐by‐state visualization.

The present research will seek to further elucidate the impact that simple shifts in visualization format may have on political perceptions. Broadly, we endeavour to determine (A) whether (and, if so, to what degree) *y*‐axis truncation of bar charts that depict intergroup political information shifts perceptions relative to bar charts with a full‐range *y*‐axis, (B) whether ‘novel’ modes of data visualization (e.g. such as those involving icon arrays)—which depict aspects of intergroup data such as response distributions and intergroup *agreement*—promote different political perceptions than more ‘traditional’ modes of presentation (i.e. bar charts) and (C) whether exposure to visualized data leads participants to hold political perceptions which differ significantly from those who are provided with no such visualizations. Should the results indicate that different modes of presentation—despite depicting identical underlying data—yield significant differences across polarization metrics, such findings would have important implications for the process by which individuals and organizations go about making visualization choices, and ultimately may provide a subtle yet highly scalable addition to the depolarization repertoire.

## METHOD

### Participants

A sample (*N* = 6603) of British (*n* = 3303) and American (*n* = 3300) participants were recruited via Ipsos MORI's internal recruitment platform. The UK sample had a mean age of 44.64, was 51.04% female and consisted of 54.50% who had obtained an educational status of NVQ4 or above. Politically, the UK sample contained 1407 respondents (i.e. 42.60%) who voted ‘Leave’ during the 2016 EU ‘Brexit’ Referendum and 1048 (i.e. 31.73%) who voted ‘Remain’. The US sample had a mean age of 46.30, was 50.82% female and consisted of 39.03% who had earned at least a Bachelor's degree. Politically, the US sample contained 1045 respondents (i.e. 31.67%) who generally consider themselves Democrats and 872 (i.e. 26.42%) who generally consider themselves to be Republican.

### Procedure

**FIGURE 1:**
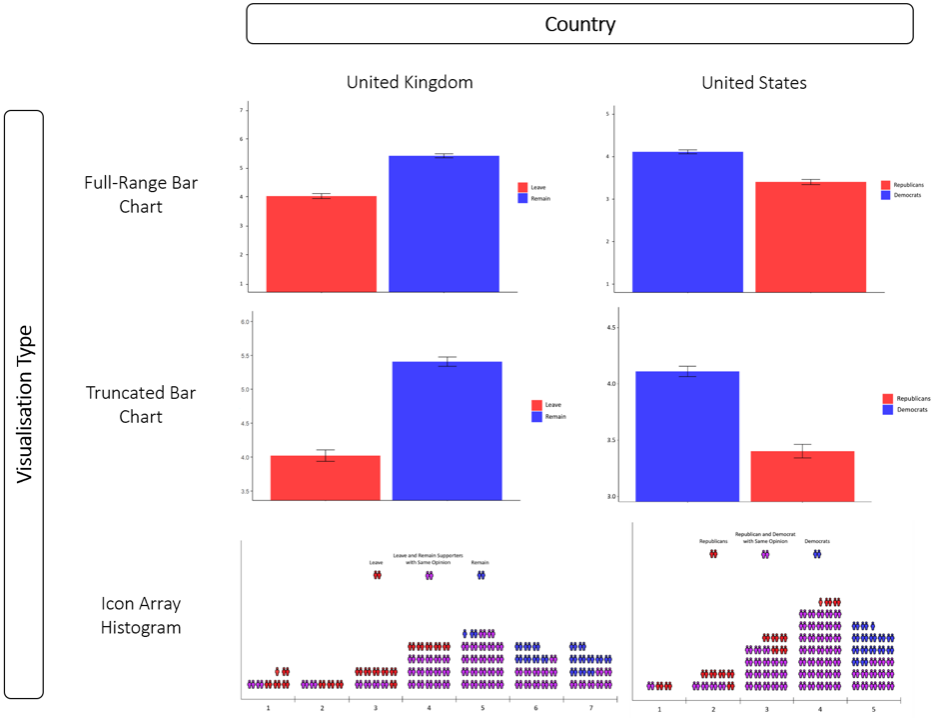
Modes of presentation used within the UK sample (left column) and US sample (right column). The top row depicts the full‐range bar charts used for each country, the middle row depicts the 1.5 bar charts used for each country and the bottom row depicts the icon array histograms used for each country.

The study utilized a between‐subjects design which featured four conditions to which participants were randomly allocated. Three conditions depicted data via the following distinct modes of presentation (see Figure 1):

1. A bar chart with a full‐range *y*‐axis (henceforth referred to simply as ‘full‐range bar chart’).
2. A bar chart with a 1.5 SD *y*‐axis (henceforth referred to simply as ‘tr bar chart’).^2^
3. An icon array histogram (essentially a set of histograms constructed using colour‐coded icons so as to more clearly highlight intergroup response overlap at each level of the survey responses; see Appendix for more detailed explanation of this novel mode).

The fourth condition—referred to as the ‘no visualization’ condition—did not visually depict the data in any way, but simply asked participants to complete questionnaire items by basing their responses on ‘what they know’ about the two groups in question (i.e. either ‘Leavers’ and ‘Remainers’ for the UK sample or Republicans and Democrats for the US sample). This no visualization condition was designed to serve as a baseline for how individuals might think about the issues devoid of any visual reference point.

The modes of presentation depicted actual data from the 2019 cross‐sectional data of the British Election Study and the 2020 American National Election Study. The items were both about immigrants' roles in the economy (see Appendix for item selection rationale). The item in the UK sample asked ‘Do you think immigration is good or bad for Britain's economy?’ whereas the item in the US sample asked, ‘How much do you agree or disagree with the following statement: Immigrants are generally good for America's economy?’

Following exposure to the randomly assigned mode of data visualization (or assignment to the no visualization condition), participants were asked to respond to a number of dependent variables (see Measures section). Additionally, participants also had their levels of general (i.e. non‐issue‐specific) affective polarization and overall perceived ideological polarization measured both pre‐ and post‐exposure to the data manipulations.

### Measures

When assessing political polarization, there are several metrics researchers may choose to employ. The most common relate to measuring the two primary types of political polarization: ideological (i.e. how far apart do groups perceive themselves to be on policy positions; e.g. see Abramowitz & Saunders, 2008) and affective (i.e. how much people ‘like’ or ‘dislike’ members of their group and the opposing group; e.g. see Iyengar et al., 2019). However, the residual impacts of political polarization may also be observed in changes in interparty dynamics such as reduced willingness to compromise (e.g. Levendusky & Malhotra, 2016; Whitt et al., 2021). Moreover, for the purposes of the present study, we are also interested in examining how shifting data visualizations may impact variables such as perceived group discreteness (a necessary component of certain types of dehumanization; Haslam, 2006) and one's responses to (and levels of agreement with) optimistic political statements (an especially relevant metric given the hostility that characterizes online political discourse).

This study's primary measures included:

*Perceived ideological polarization*: Participants were asked to indicate their response (via a 100‐point scale that ranged from ‘Not at All Polarizing’ and ‘Extremely Polarizing’) to the item ‘Based on the graph, how polarizing do you believe this issue is for the two groups?’

*Perceived affective polarization*: Participants were asked to indicate their response (via a 100‐point scale that ranged from ‘Extremely Cold/Negative’ and ‘Extremely Warm/Positive’) to the item ‘Based on the graph, how warm (i.e. positive) or cold (i.e. negative) do you think members of each group feel towards members of the other group on this issue?’

*Estimates of intergroup opinion overlap*: To determine how modes impact the *accuracy* of participant views of intergroup agreement, we asked participants to estimate intergroup opinion overlap by indicating their response (via a 100‐point scale) to the item ‘Based on the graph, what percentage of the two groups share the same opinion on the issue?’ The actual levels of overlap (and thus, the benchmarks for these responses) are covered in the Results section.

*Level of intergroup agreement relative to expectations*: Participants were asked to indicate their response (via a 5‐point scale that ranged from ‘They seem to agree much less than I would have thought’ to ‘They seem to agree much more than I would have thought’) to the item ‘Based on the graph, how would you classify the level of agreement between the two groups on this issue?’

*Perceived group discreteness*: Participants were asked to indicate their response (via a 6‐point scale that ranged from ‘Strongly Disagree’ to ‘Strongly Agree’) to the item ‘Based on the graph, how much do you agree with the following statement: when it comes to this issue, [“Leavers” and “Remainers”/Republicans and Democrats] just seem to be two completely different types of people?’

*Perceived likelihood of intergroup compromise*: Participants were asked to indicate their response (via a 100‐point scale that ranged from ‘Not at All Likely’ and ‘Extremely Likely’) to the item ‘Based on the graph, how likely do you think it is that [“Leavers” and “Remainers”/Republicans and Democrats] could find ways to compromise on this issue?’

*Agreement with optimistic political statement*: Participants were presented with the following statement and asked to indicate, via open‐text response (which would later be blind‐coded on a 1–5 scale by two independent raters), the degree to which they agreed or disagreed with the sentiment expressed:

Imagine you came across the following comment in one of your networks:

Listen, [“Leavers” and “Remainers”/Republicans and Democrats] are definitely different – I get that. They disagree on a lot and they'll probably always argue over the right way to do things. But I actually think – if we look past some of the more extreme members of each group – we'd probably find that we're not as far apart on some issues than a lot of people think. I don't think we'll ever be fully in agreement on everything, but I do think if we made more of an effort to look for what unites us as opposed to what divides us, we might find areas where we can compromise and cooperate.

### Hypotheses

Table 1 below depicts the hypotheses^3^ for the study.

**TABLE 1:** Study hypotheses.

Label	Hypothesis
H1	Mode of presentation will have a significant main effect on issue‐specific perceptions of ideological polarization but will not have a significant main effect on issue‐specific affective polarization. For both measures of polarization, all three modes will promote significantly lower perceptions of both ideological and affective polarization relative to the no visualization condition
H2	Mode of presentation will have a significant main effect on levels of intergroup agreement relative to expectations. The full‐range bar chart and the icon array histogram will promote significantly higher levels of agreement relative to expectations (i.e. participants will be more likely to report that the groups “appear to agree more than they expected”) compared to the truncated bar chart
H3	Mode of presentation will have a significant main effect on perceived group “discreteness.” The full‐range bar chart and the icon array histogram will promote significantly lower levels of perceived discreteness compared to the no visualization condition, while no significant differences will emerge between the truncated bar chart and no visualization condition
H4	Mode of presentation will have a significant main effect on perceptions of likelihood of intergroup compromise. All three modes will promote significantly higher levels of compromise likelihood than the no visualization condition
H5	The full‐range bar chart and the icon array histogram will promote estimates of intergroup opinion overlap that will be more accurate than the estimates produced within the 1.5 S bar chart and no visualization conditions
H6	Mode of presentation will have a significant main effect on pre‐ and post‐exposure measures of general ideological polarization, but will not have a significant main effect on pre‐ and post‐exposure measures of general affective polarization

## RESULTS

An overview of results can be found in Table 2 (for the UK sample) and Table 3 (for the US sample) below:

**TABLE 2:** Main effects of mode of data visualization (United Kingdom sample).

Outcome measure	No visualization		Truncated bar chart		Full‐range bar chart		Icon array histogram		*F*	$\eta_{p}^{2}$	*p*
	*M*	SD	*M*	SD	*M*	SD	*M*	SD			
Perceptions of issue‐specific ideological polarization	60.45	24.73	60.22	22.30	55.34	20.91	56.29	22.51	11.200 (3, 3299)	.010	<.001
Perceptions of issue‐specific affective polarization	55.82	24.51	55.54	23.60	51.91	23.80	54.14	22.68	4.706 (3, 3299)	.004	.003
Change in overall perceived ideological polarization	−0.21	19.19	−0.75	19.44	−4.83	20.91	−4.53	21.61	11.890 (3, 3299)	.011	<.001
Change in general affective polarization	−6.10	28.45	−8.83	30.70	−6.29	31.29	−8.69	28.24	1.539 (3, 2451)	.002	.202
Estimates of intergroup opinion overlap	43.59	23.98	44.55	21.49	51.46	19.82	54.71	19.84	52.600 (3, 3299)	.046	<.001
Intergroup agreement relative to expectations	NA	NA	2.92	1.01	3.24	1.02	3.38	1.05	42.101 (2, 2475)	.033	<.001
Perceptions of intergroup discreteness	4.33	1.19	4.20	1.11	3.72	1.14	3.73	1.22	61.180 (3, 3299)	.053	<.001
Beliefs about compromise likelihood	43.65	23.06	43.31	21.62	51.73	21.37	51.25	21.77	36.700 (3, 3299)	.032	<.001
Response to optimistic political statement	3.48	1.58	3.61	1.53	3.80	1.46	3.66	1.51	6.441 (3, 3298)	.006	<.001

**TABLE 3:** Main effects of mode of data visualization (United States sample).

Outcome measure	No visualization		Truncated Bar chart		Full‐range bar chart		Icon array histogram		*F*	$\eta_{p}^{2}$	*p*
	*M*	SD	*M*	SD	*M*	SD	*M*	SD			
Perceptions of issue‐specific ideological polarization	59.68	26.54	58.01	25.59	52.57	25.58	54.66	24.54	13.020 (3, 3296)	.012	<.001
Perceptions of issue‐specific affective polarization	56.75	25.86	57.24	26.10	55.41	25.07	53.66	24.43	3.276 (3, 3296)	.003	.020
Change in overall perceived ideological polarization	−1.07	21.04	−2.12	21.71	−5.05	21.64	−2.58	23.48	4.862 (3, 3296)	.004	.002
Change in general affective polarization	−5.68	26.16	−1.78	24.41	−4.89	22.28	−4.13	21.63	2.366 (3, 1856)	.004	.069
Estimates of intergroup opinion overlap	42.59	25.40	43.51	22.62	54.27	23.58	54.46	23.00	63.070 (3, 3296)	.054	<.001
Intergroup agreement relative to expectations	NA	NA	3.01	1.13	3.42	1.24	3.47	1.24	36.460 (3, 2472)	.029	<.001
Perceptions of intergroup discreteness	4.58	1.27	4.46	1.21	3.71	1.32	3.88	1.36	90.800 (3, 3296)	.076	<.001
Beliefs about compromise likelihood	41.35	24.70	41.39	23.81	51.07	24.95	50.04	25.54	38.120 (3, 3296)	.034	<.001
Response to optimistic political statement	3.47	1.63	3.60	1.57	3.78	1.50	3.71	1.58	6.044 (3, 3296)	.005	<.001

**FIGURE 2:**
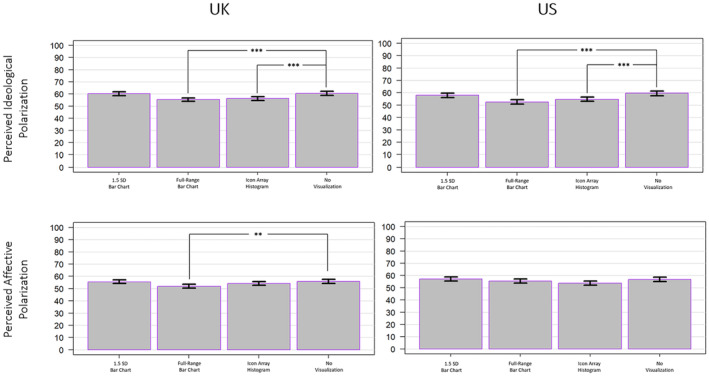
Perceptions of ideological polarization (top row) and affective polarization (bottom row) by mode for the UK sample (left column) and the US sample (right column). * *p* < .05, ** *p* < .01, *** *p* < .001.

Results for the one‐way ANOVA for perceived ideological polarization indicated a significant main effect of mode of presentation on perceptions of ideological polarization for both the UK sample (*F*(3, 3299) = 11.2, *p* < .001, $\eta_{p}^{2}$ = .010) as well as for the US sample (*F*(3, 3296) = 13.02, *p* < .001, $\eta_{p}^{2}$ = .012). For the UK sample, Tukey HSD post hoc tests determined that exposure to the full‐range bar chart and icon array histogram promoted perceptions of ideological polarization that differed significantly from the no visualization condition (*p* < .001 and *p* = .001, respectively), but exposure to the 1.5 S bar chart did not (*p* = .997). For the US sample, Tukey HSD post hoc tests determined that exposure to the full‐range bar chart and icon array histogram promoted perceptions of ideological polarization that differed significantly from the no visualization condition (*p* < .001 and *p* < .001, respectively), but exposure to the 1.5 SD bar chart did not (*p* = .543). Results of the one‐way ANOVA for affective polarization also indicated a significant main effect of mode of presentation for both the UK sample (*F*(3, 3299) = 4.706, *p* = .003, $\eta_{p}^{2}$ = .004) as well as for the US sample (*F*(3, 3296) = 3.276, *p* = .020, $\eta_{p}^{2}$ = .003), although with a smaller effect size than that of the effects of mode on perceived ideological polarization (as measured by partial η^2^; see Figure 2). For the UK sample, Tukey HSD post hoc tests determined that exposure to the full‐range bar chart promoted perceptions of affective polarization that differed significantly from the no visualization condition (*p* = .004), but exposure to the icon array histogram and the truncated bar chart did not (*p* = .472 and *p* = .995, respectively). For the US sample, Tukey HSD post hoc tests determined that neither exposure to the full‐range bar chart, the icon array histogram, nor the bar chart promoted perceptions of affective polarization that differed significantly from the no visualization condition (*p* = .705, *p* = .065, and *p* = .980, respectively).

To determine whether the issue‐specific effects of mode extended to more general measures of polarization, differences between the pre‐ and post‐measures of overall (i.e. non‐issue‐specific) perceived ideological polarization and affective polarization were calculated to determine whether changes varied as a function of condition.

**FIGURE 3:**
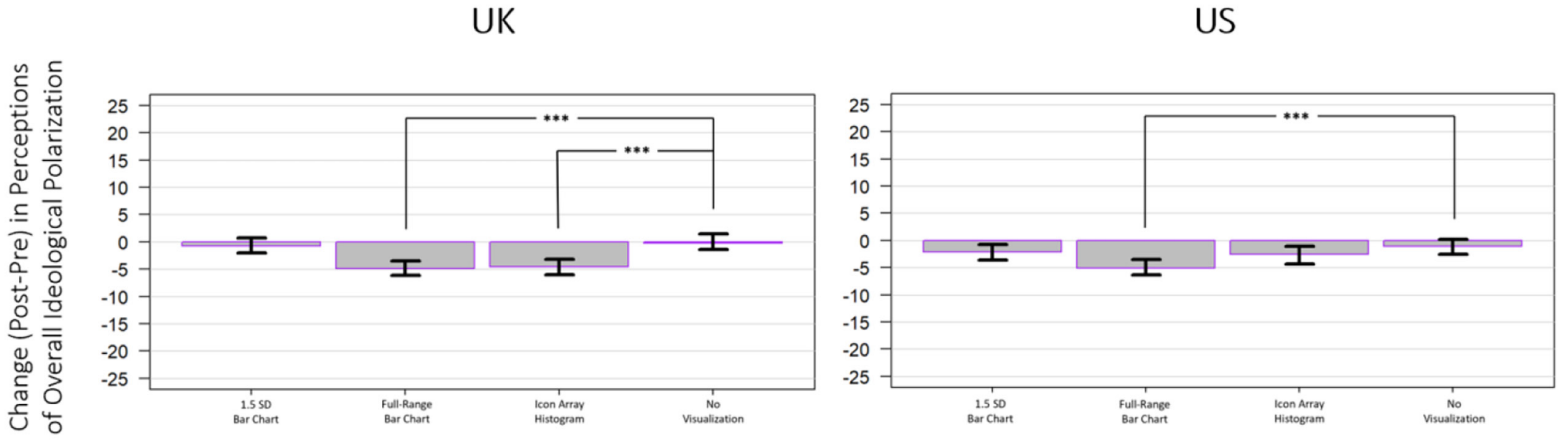
Change in the pre‐ and post‐measurements of perceived overall ideological polarization by mode for the UK sample (left) and the US sample (right). * *p* < .05, ** *p* < .01, *** *p* < .001.

A one‐way ANOVA revealed significant main effects of mode of presentation for changes (i.e. post–pre) in overall perceived issue polarization for both the UK sample (*F*(3, 3299) = 11.89, *p* < .001, $\eta_{p}^{2}$ = .011) and the US sample (*F*(3, 3296) = 4.862, *p* = .002, $\eta_{p}^{2}$ = .004; see Figure 3).

**FIGURE 4:**
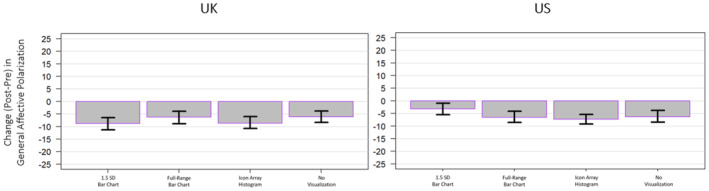
Change in the pre‐ and post‐measurements of general affective polarization by mode for the UK sample (left) and the US sample (right).

A one‐way ANOVA on changes in self‐reported general affective polarization pre‐ and post‐mode exposure for self‐identified Leavers and Remainers in the UK (a subsample of 2455) revealed no significant change in overall affective polarization (*F*(3, 2451) = 1.539, *p* = .202, $\eta_{p}^{2}$ = .002). In the US, pre‐ and post‐measures of general affective polarization for Democrats and Republicans (a sub‐sample of 1860) revealed no significant change in overall affective polarization based on a one‐way ANOVA of the difference in pre–post scores (*F*(3, 1856) = 2.366, *p* = .069, $\eta_{p}^{2}$ = .004; see Figure 4).

**FIGURE 5:**
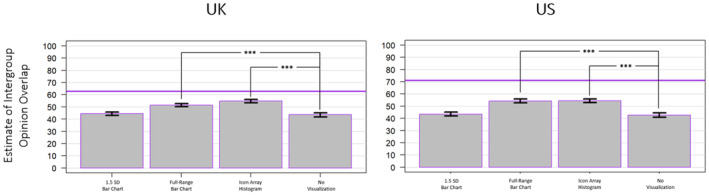
Estimates of intergroup opinion overlap by mode for the UK sample (left) and the US sample (right). The horizontal purple line indicates the actual level of overlap for the data used in each sample. * *p* < .05, ** *p* < .01, *** *p* < .001.

An exploratory one‐way ANOVA on estimates of intergroup opinion overlap revealed a significant main effect of mode of presentation for both the UK sample (*F*(3, 3299) = 52.6, *p* < .001, $\eta_{p}^{2}$ = .046) and the US sample (*F*(3, 3296) = 63.07, *p* < .001, $\eta_{p}^{2}$ = .054; see Figure 5). However, beyond main effects (which can only detect whether the modes differ significantly *from one another*), the modes were also assessed relative to an accuracy benchmark known as *percentage of common scores* (PCS; Hanel et al., 2019). For the UK data, the PCS was approximately 63%; for the US data, the PCS was approximately 71%. Across both samples, all four conditions yielded estimates that fell significantly below their respective accuracy benchmarks. However, the full‐range bar chart and the icon array histogram (but not the 1.5 SD bar chart) elicited estimates that differed significantly from the no visualization condition in the desired direction (i.e. more closely approaching the accuracy benchmark) in both the UK (*t*(1591.6) = 7.271, *p* < .001, *d* = 0.36 and *t*(1592.6) = 10.27, *p* < .001, *d* = 0.51, respectively) and the US (*t*(1639.7) = 9.686, *p* < .001, *d* = 0.48 and *t*(1632) = 9.951, *p* < .001, *d* = 0.49, respectively) samples.

**FIGURE 6:**
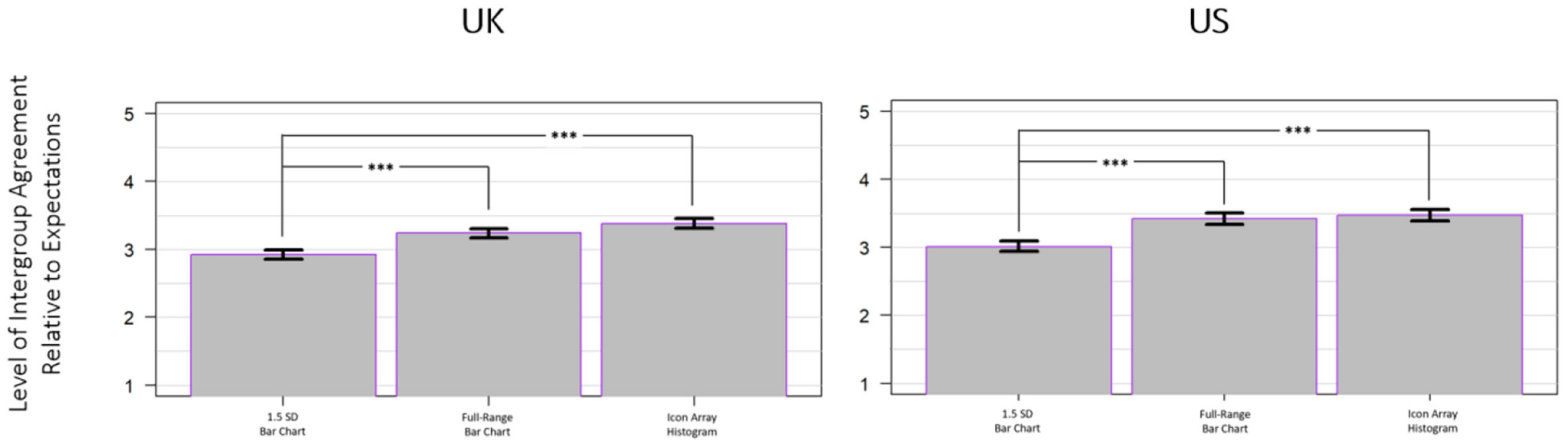
Perceptions concerning levels of intergroup agreement relative to participant expectations by mode for the UK sample (left) and the US sample (right). Higher responses correspond to perceived levels of agreement greater than what participants expected. * *p* < .05, ** *p* < .01, *** *p* < .001.

A one‐way ANOVA was conducted to assess the main effect of mode of presentation on level of intergroup agreement relative to expectations, which revealed a significant main effect for both the UK sample (*F*(2, 2475) = 42.01, *p* < .001, $\eta_{p}^{2}$ = .033) and US sample (*F*(2, 2472) = 36.46, *p* < .001, $\eta_{p}^{2}$ = .029; see Figure 6). For the UK sample, Tukey HSD post hoc tests determined that exposure to the full‐range bar chart and icon array histogram promoted greater perceptions of intergroup agreement relative to expectations than the 1.5 SD bar chart condition (*p* < .001 and *p* < .001, respectively). For the US sample, Tukey HSD post hoc tests also determined that exposure to the full‐range bar chart and icon array histogram promoted greater perceptions of intergroup agreement relative to expectations than the 1.5 SD bar chart condition (*p* < .001 and *p* < .001, respectively).

**FIGURE 7:**
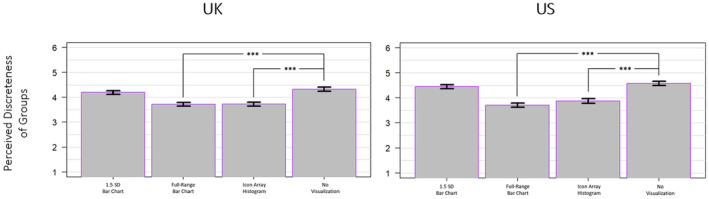
Perceptions of group discreteness by mode for the UK sample (left) and the US sample (right). * *p* < .05, ** *p* < .01, *** *p* < .001.

A one‐way ANOVA was conducted to assess the main effect of mode of presentation on perceived group discreteness, which revealed a significant main effect for both the UK sample (*F*(3, 3299) = 61.18, *p* < .001, $\eta_{p}^{2}$ = .053) and the US sample (*F*(3, 3296) = 90.8, *p* < .001, $\eta_{p}^{2}$ = .076; see Figure 7). For the UK sample, Tukey HSD post hoc tests determined that exposure to the full‐range bar chart and icon array histogram promoted significantly lower perceptions of intergroup discreteness than the no visualization condition (*p* < .001 and *p* < .001, respectively) but exposure to the 1.5 SD bar chart did not (*p* = .134). For the US sample, Tukey HSD post hoc tests determined that exposure to the full‐range bar chart and icon array histogram promoted significantly lower perceptions of intergroup discreteness than the no visualization condition (*p* < .001 and *p* < .001, respectively) but exposure to the 1.5 SD bar chart did not (*p* = .236).

**FIGURE 8:**
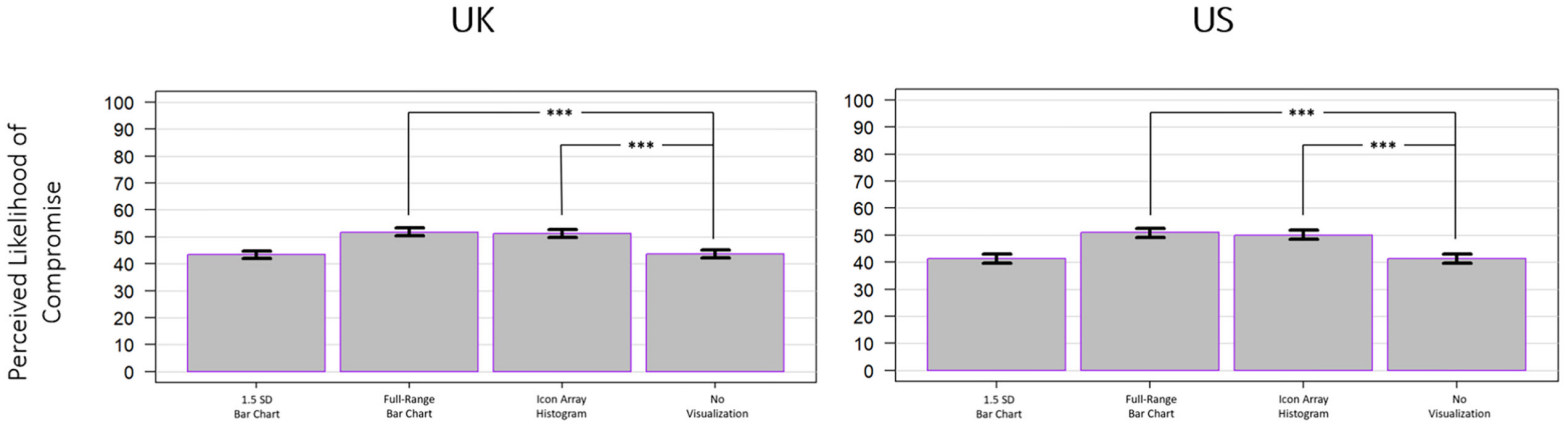
Perceptions of the likelihood of compromise by mode for the UK sample (left) and the US sample (right). * *p* < .05, ** *p* < .01, *** *p* < .001.

A one‐way ANOVA was conducted to assess the main effect of mode of presentation on the perceived likelihood of intergroup compromise, which revealed a significant main effect for both the UK sample (*F*(3, 3299) = 36.7, *p* < .001, $\eta_{p}^{2}$ = .032) and the US sample (*F*(3, 3296) = 38.12, *p* < .001, $\eta_{p}^{2}$ = .034; see Figure 8). For the UK sample, Tukey HSD post hoc tests determined that exposure to the full‐range bar chart and icon array histogram promoted significantly higher perceptions of compromise likelihood than the no visualization condition (*p* < .001 and *p* < .001, respectively) but exposure to the 1.5 SD bar chart did not (*p* = .990). For the US sample, Tukey HSD post hoc tests determined that exposure to the full‐range bar chart and icon array histogram promoted significantly higher perceptions of compromise likelihood than the no visualization condition (*p* < .001 and *p* < .001, respectively) but exposure to the 1.5 SD bar chart did not (*p* > .999).

**FIGURE 9:**
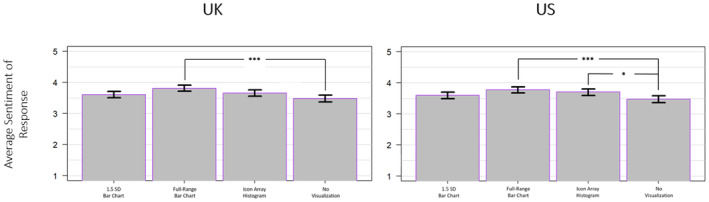
Average sentiment score following exposure to an optimistic political statement by mode for the UK sample (left) and the US sample (right). * *p* < .05, ** *p* < .01, *** *p* < .001.

Finally, an exploratory one‐way ANOVA was conducted to assess whether the main effects of mode would be observed on participant response valence to an optimistic political statement. To run the analysis, open‐ended participant responses were manually coded (on a 1 to 5 scale) by two third‐party raters blind to the conditions of the sample. This analysis revealed a significant main effect for the UK sample (*F*(3, 3298) = 6.441, *p* < .001, $\eta_{p}^{2}$ = .006) as well as for the US sample (*F*(3, 3296) = 6.044, *p* < .001, $\eta_{p}^{2}$ = .005; see Figure 9).

## DISCUSSION

The results demonstrate that perceptions of polarization are not fixed and can be influenced by the mode in which data about group differences are graphically represented. In both the UK and the US, the mode of presentation had a significant impact on perceptions of how ideologically polarizing the issue seemed to be, how affectively polarized the two groups were likely to be on the issue, pre‐ and post‐measures of perceived overall ideological polarization, perceptions of intergroup opinion overlap, perceptions of intergroup agreement relative to expectations, perceptions of group discreteness, perceptions of the likelihood of compromise and expressions of positive sentiment in response to political optimism. The most robust results concerned mode's impact on estimates of intergroup opinion overlap, perceived intergroup agreement relative to expectations, perceived group discreteness and perceptions of the likelihood of compromise.

Importantly, the findings indicate that certain modes consistently yield more desirable results than others. More specifically, the full‐range bar chart and the icon array histogram routinely reduced polarized perceptions, increased the accuracy of intergroup opinion overlap estimates and decreased perceptions of group discreteness relative to both the 1.5 SD bar chart and the no visualization condition. Such insight is critical as it implies that deliberate choices related to data visualization can produce meaningful shifts in partisan perceptions. For example, in the US, the choice to not depict data and to simply allow individuals to make assumptions based on their prior knowledge of the issue (i.e. whether immigrants are generally good for America's economy) as opposed to utilizing a full‐range bar chart to depict the data would result, on average, in a 12.67%^4^ difference in perception of issue‐specific ideological polarization, 24.13% difference in estimates of intergroup opinion overlap, a 20.98% difference in perceptions of group discreteness and a 21.05% difference in estimates of compromise likelihood—all of which represent highly significant statistical differences. Relatedly, it is not just the choice between including a visualization versus not including one that proves consequential; a ‘poor’ choice of mode of data presentation (vs. a more sensible one) can also have profound effects on partisan perceptions and beliefs. For instance, if an agency in the UK were to opt for a 1.5 SD bar chart as opposed to an icon array histogram to depict the polarized issue featured within the study, such a choice would result, on average, in a 6.74% difference in perceptions of issue‐specific ideological polarization, a 20.48% difference in estimates of intergroup opinion overlap, an 11.96% difference in perceptions of group discreteness and a 16.78% difference in estimates of compromise likelihood.

Not only does the current study suggest that perceptions of levels of polarization are malleable (even for issues where degrees of polarization may be historically entrenched), but it also shows that perceptions of *political groups themselves* are malleable. Our findings suggest that mode of presentation can also moderate the degree to which we see political opponents as discrete and thus fundamentally different from one another. Such a finding should not be overlooked as the perceived discreteness of groups has been implicated in prior literature as a contributor to essentialist beliefs (Haslam et al., 2004), which appear to be a necessary component for certain forms of dehumanization (Haslam, 2006). Consequently, the ability of certain modes to mitigate perceptions of discreteness may prove to be a subtle yet valuable lever by which agents may be able to prevent the exacerbation of interparty hostility and alter the trajectory of intergroup relations.

**FIGURE 10:**
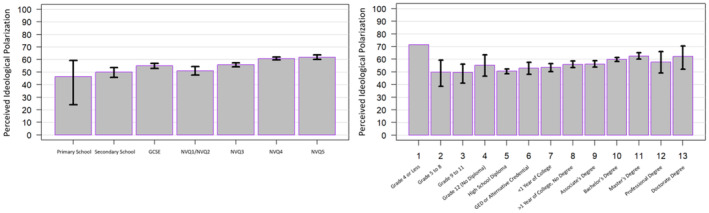
Perceptions of ideological polarization by educational attainment for the UK sample (left) and the US sample (right).

Moreover, the study also offered a unique, serendipitous insight. Exploratory analyses revealed that perceptions of ideological polarization were greatest for participants with the most education (see Appendix) (Figure 10). While such a finding may offer a modest contribution to the literature on the relationship between education and polarization, we believe the more relevant takeaway for the current study is that, if overestimations of polarization are being disproportionately driven by the highly educated, and should this group be more likely to consume certain types of news (Schulz et al., 2019), perhaps they would be more likely to encounter interventions such as the one being proposed here (i.e. embedding ‘better’ visualizations of inter‐party data into communications). It would be important to test whether interventions which embed ‘better’ visualizations across these news and polling platform media could yield stronger effects when sustained over time.

The current study provides a framework by which any media outlet, polling organization, government agency or any other political actor interested in reducing polarized perceptions can implement a simple yet effective intervention. Moreover, the potential scalability of this intervention may make it a more defensible candidate for widespread application than other options found in the academic literature. Though perceptions of polarization are one step removed from actual polarization, scholars in the field have argued that the two processes might be self‐reinforcing (Lees & Cikara, 2021), lending greater urgency to the need to curtail exaggerated beliefs about political others. However, we strongly suspect the implications of this research may also extend beyond the domain of political polarization. The issue of persistent, seemingly intractable disagreement is not exclusive to politics. Factions may—and often do—form both within and between organizations and institutions based on disputes stemming from divergent philosophies or incompatible beliefs. Should such disputes share similar dynamics to political polarization, *perceptions* of disagreement may outpace the *actual levels* of disagreement, but may also, critically, create a self‐reinforcing cycle wherein mere perceptions may ultimately drive the true progression of the divide. Ultimately, over time, differences of opinion risk becoming ‘essentialized’, at which point the motivation for reconciliation suffers as groups come to view one another as *fundamentally* different. The current research finds that certain modes (such as truncated bar charts) may do little to disrupt the momentum of this cycle and often simply provide justification for the antagonism via depictions which support the irreconcilable disagreement narrative while bolstering the perceived ‘otherness’ of the opposition. However, modes such as full‐range bar charts and icon array histograms, via their non‐exaggerated depiction of group differences and more balanced coverage of group agreement, respectively, may be capable of violating expectations regarding the extent of group differences. This violation may effectively disrupt the cyclical escalation of intergroup animus, permitting groups the opportunity to reassess their actual levels of disagreement, acknowledge their (often substantial but under‐emphasized) areas of agreement, and potentially shift the trajectory of the relationship in a more positive direction.

While the current study highlights promising evidence for the role of mode of presentation in shifting perceptions of polarization, it is not without its limitations. Firstly, while mode was shown to be capable of shifting myriad intergroup perceptions relevant to polarization (including multiple measures of polarization itself), the current study provides only weak evidence that it might be capable of impacting political sentiment. Such a finding is consistent with our prior research, which found little evidence to suggest that mode exerts a significant impact on partisan behaviour, and inconsistent evidence for the longevity of the effects of the single‐exposure intervention on perceptions over a multi‐week time period. While we believe that shifting perceptions is undoubtedly an important step, more work is required, both in academia and in partnership with real‐world stakeholders, to determine whether (and, if so, to what degree) the effects of mode of presentation might extend beyond perceptions to impact partisan behaviour, and whether shifts in perception may become more permanent when repeated‐exposure paradigms are employed. Secondly, many of the outcome variables used in this investigation contained the phrase ‘based on the data’, explicitly encouraging participants use the visualizations to inform their responses. While this was an intentional methodological choice on our part, it is not unreasonable to assume that the effects observed may have been influenced by this deliberate refocusing of participant attention. Future research should seek to determine whether similar visualization effects are obtained without the need to provide such overt cues. Thirdly, while some mode effects were pronounced, the interventions were not longitudinal in nature, and thus future research should investigate their long‐terms effects and rates of decay. Finally, it will be important for future research to establish how these effects fare in a ‘competitive’ message environment (see Bolsen & Shapiro, 2017) where other political actors might be trying to shift perceptions in *more* polarized directions by emphasizing group differences.

## CONCLUSION

Overall, the present study confirmed that changing mode of presentation—a simple and highly scalable intervention—is capable of exerting significant influence on an array of critical interparty perceptions and beliefs, even on a highly polarized issue for which individuals ostensibly hold strong attitudinal priors.

Between partisan ‘echo chambers’ (Terren & Borge‐Bravo, 2021), selective, ideologically‐aligned media exposure (Stroud, 2011) and the rapid emergence of new, polarized and otherwise hostile information ecosystems online, it is reasonable to assume that individuals might have disproportionate exposure to biased data. Truncated bar charts, by virtue of their ability to visually accentuate group *differences*, are a commonly used method of data presentation (Hanel et al., 2019) and may also constitute a depiction of political data more commonly used in such ecosystems, and thus more congruent with participant expectations. Conversely, the full‐range bar chart's *reduced* exaggeration of group differences and the icon array histogram's inclusion of distributional and similarity information may induce expectation violations and surprise, which can play an important role in belief updating (e.g. Nassar et al., 2010). Ultimately, we believe that many of the results contained herein may be products of the interaction between participant *expectations* about what intergroup agreement *should* look like (informed by inputs which tend to highlight disagreement in a highly polarized environment) and what inter‐group data depicted in less biased, more balanced ways actually *does* look like.

While further work is needed to determine the extent and boundary conditions of the effects of mode (particularly as they relate to ‘real‐world’ behaviour), the evidence presented clearly demonstrates that those who disseminate data about political groups should choose their visualizations carefully and deliberately, as the manner in which the data are presented plays a significant role in a multitude of inter‐group‐relevant perceptions and attitudes.

## AUTHOR CONTRIBUTIONS

**JonRobert Tartaglione:** Conceptualization; methodology; writing – original draft; data curation; investigation; funding acquisition; formal analysis; project administration; writing – review and editing. **Lee de‐Wit:** Conceptualization; methodology; writing – review and editing; funding acquisition; project administration; supervision.

## CONFLICT OF INTEREST STATEMENT

The authors declare no conflict of interest.

## Data Availability

The data that support the findings of this study are available from Ipsos MORI. Restrictions apply to the availability of these data, which were used under license for this study. Data are available from the author(s) with the permission of Ipsos MORI.

## Footnotes

^1^
More recently, icon arrays have even shown themselves capable of assisting in pressing, real‐world issues such as a reticence to get vaccinated against COVID‐19 by reducing the concern over exceedingly rare side effects of vaccines (Fansher et al., 2022).

^2^
Some researchers have argued that 1.5 standard deviations of the grand mean of the data might represent a degree of truncation that promotes an appropriate sensitivity to effect size (Witt, 2019).

^3^
A link to the OSF preregistration can be found here: https://osf.io/yzq5w.

^4^
Differences were calculated by taking the absolute value of the change in value (e.g. between the full‐range bar chart measure and the no visualization measure), dividing it by the average of the two figures and multiplying this figure by 100.

## Constructing Icon array histograms

Icon array histograms are essentially superimposed histograms constructed by using icons as the building blocks. Our icon array histograms relied primarily on ‘paired icons’, which are two ‘restroom icons’,
^5^
One of the more niche findings within the icon array literature concerns the type of icon used. Zikmund‐Fisher et al. (2014) tested a number of icon types (e.g. blocks, ovals, etc.) to determine whether the choice of symbols used plays a role in how the information is processed and retained. Overall, icon type was found to play a significant role in perception, retention and recall of risk, with ‘restroom icons’—the, minimalist, gendered silhouettes traditionally used to differentiate men's restrooms from women's—to be especially promising. joined side‐by‐side to represent the opinion of two separate respondents. For each bin, we first make as many cross‐party pairs as possible. For example, if 10 members of Group A gave a response of one on a Likert scale, and six members of Group B gave that same response, then we would be able to make six cross‐party pairs. This would leave a surplus of four Group A respondents in that response bin, who would then become two same‐party pairs. Thus, that response bin would contain six cross‐party pairs and two same‐party pairs, representing all 16 individuals who offered that response. This matching pairs system permits icon array histograms to highlight ‘overlap’ (i.e. where members of two groups are providing the same response) more effectively than traditional modes of presentation (e.g. bar charts), which often offer minimal distributional information, while also representing the distribution of responses in a faithful and accurate manner.

## Rationale for selecting immigration as the issue presented to participants

The particular items used in the study were selected for a number of reasons. Firstly, our original series of studies began by using artificial datasets that depicted intergroup data with an effect size (i.e. Cohen's *D*) of precisely 0.80 (i.e. the commonly accepted threshold for an effect to be categorized as ‘large’). As we moved from artificial data to actual data in our earlier studies, we sought to identify a policy area that typically yielded intergroup (i.e. between Republicans and Democrats) effect sizes that hovered around the 0.80 range. Based on prior ANES data, immigration seemed to be a satisfactory fit for that criterion. Similarly, the data from the US immigration item used in the present study has a Cohen's *D* of 0.72. While the UK immigration item has a slightly higher Cohen's *D* (i.e. 0.90 when dividing respondents into ‘Leavers’ and ‘Remainers’), we felt the difference was justifiable, especially since utilizing nearly identical items for both countries would make cross‐cultural comparisons more useful.

## Interaction between education and perceived issue polarization

Though not originally planned, an exploratory ANOVA for perceived ideological polarization which included both mode and educational attainment as independent variables also found the latter to exert significant main effects for both the UK sample and the US sample, such that greater educational attainment corresponded with higher levels of perceived ideological polarization (see Figure 10).
